# 4:10 Cycles of Very Low Calories Protect Against Tumor Xenografts, but Not Metastases in the Absence of Chemotherapy

**DOI:** 10.1093/geroni/igaa057.402

**Published:** 2020-12-16

**Authors:** Laura Corrales-Diaz Pomatto, Oye Bosompra, Sarah Wong, Monica Bodogai, Jonathan Kato, Melissa Carpenter, Arya Biragyn, Rafael de Cabo

**Affiliations:** National Institute on Aging, Bethesda, Maryland, United States

## Abstract

Cancer is a leading cause of mortality, with its incidence only expected to rise with an increasingly aging population. Dietary interventions, primarily caloric restriction (CR), lower cellular energy metabolism and have long been utilized to slow the aging process and protect against age-related diseases, including cancer. However, due to the stringency of CR, dietary alternatives that offer the same beneficial outcomes in cancer prevention and longevity have become increasingly attractive. Periodic cycles (4 days twice a month) of low caloric intake followed by a standard ad libitum (AL) diet was previously shown to promote health-span in mice and humans and protect against primary tumorigenesis and enhanced the effects of chemotherapy. The aim of our study was to compare the tumorigenic potential of 4T1 cells, a murine model of stage IV breast cancer, in young and aged female BALB/c mice fed either periodic cycles of low caloric diets versus chronic 20% CR. Compared to AL controls, we found a significant delay in primary tumor growth in mice regardless of diet composition by the 4:10 cycles of very low caloric intake. However, unlike in CR, CR-alternative diets were not protective against lung metastases in the absence of chemotherapy. Our study sheds light into the underlying differences of calorie-based interventions in the absence of chemotherapy.

